# Similarities and differences between study designs in short‐ and long‐term outcomes of laparoscopic versus open low anterior resection for rectal cancer: A systematic review and meta‐analysis of randomized, case‐matched, and cohort studies

**DOI:** 10.1002/ags3.12409

**Published:** 2020-11-21

**Authors:** Nobuaki Hoshino, Yudai Fukui, Koya Hida, Kazutaka Obama

**Affiliations:** ^1^ Department of Surgery Kyoto University Graduate School of Medicine Kyoto Japan

**Keywords:** case‐matched study, low anterior resection, randomized controlled trial, rectal cancer

## Abstract

**Aim:**

Randomized controlled trials (RCT) are the gold standard in surgical research, and case‐matched studies, such as studies with propensity score matching, are expected to serve as an alternative to RCT. Both study designs have been used to investigate the potential superiority of laparoscopic surgery to open surgery for rectal cancer, but it remains unclear whether there are any differences in the findings obtained using these study designs. We aimed to examine similarities and differences between findings from different study designs regarding laparoscopic surgery for rectal cancer.

**Methods:**

Systematic review and meta‐analyses. A comprehensive literature search was conducted using PubMed, Scopus, and Cochrane. RCT, case‐matched studies, and cohort studies comparing laparoscopic low anterior resection and open low anterior resection for rectal cancer were included. In total, 8 short‐term outcomes and 3 long‐term outcomes were assessed. Meta‐analysis was conducted stratified by study design using a random‐effects model.

**Results:**

Thirty‐five studies were included in this review. Findings did not differ between RCT and case‐matched studies for most outcomes. However, the estimated treatment effect was largest in cohort studies, intermediate in case‐matched studies, and smallest in RCT for overall postoperative complications and 3‐year local recurrence.

**Conclusion:**

Findings from case‐matched studies were similar to those from RCT in laparoscopic low anterior resection for rectal cancer. However, findings from case‐matched studies were sometimes intermediate between those of RCT and unadjusted cohort studies, and case‐matched studies and cohort studies have a potential to overestimate the treatment effect compared with RCT.

## Graphical abstract

This article reports on similarities and differences in findings of randomized controlled trials (RCTs), case‐matched studies, and cohort studies, as revealed by meta‐analyses of previously published studies on laparoscopic low anterior resection (LAR) for rectal cancer. Results of case‐matched studies were often similar to those of RCTs in terms of outcomes of laparoscopic LAR for rectal cancer. However, case‐matched studies occasionally overestimate the effects of interventions compared to RCTs.

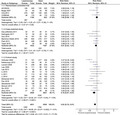

## 1 INTRODUCTION

Observational studies inherently include confounding factors that can affect their results, and several differences have been noted between the findings of randomized controlled trials (RCT) and those of observational studies^1^. For adequate comparison of interventions in observational studies, matching methods are frequently used in surgical research and the most common method is propensity score matching^2^. First introduced by Rosenbaum et al, propensity score matching is expected to be an alternative to RCT^3^, ^4^, which are currently considered the gold standard in surgical research investigating the treatment effect of an intervention. Although it may be favorable to conduct RCT, this is often difficult to do for practical and ethical reasons in surgical research^2^. In addition, RCT generally require substantial resources including time, money, and collaboration among diverse specialists in order to ensure patient safety, data correctness, and standardization of interventions.

Recently, nationwide databases such as the National Clinical Database (NCD) and the National Database (NDB) have become available, despite several restrictions on their use^5^, ^6^. If findings from case‐matched studies are similar to those from RCT, case‐matched studies using a nationwide database may supersede RCT. There are methodological differences between RCT and case‐matched studies such as patient selection and adjustment for confounding factors^7^, ^8^. However, it remains unclear whether there are differences in findings between RCT and case‐matched studies.

We aimed to investigate similarities and differences in findings between RCT, case‐matched studies, and cohort studies regarding laparoscopic low anterior resection (LAR) for rectal cancer. LAR is defined as a procedure representing the performance of surgery in NCD^5^. These study designs have been used to investigate the potential superiority of laparoscopic LAR to open LAR for rectal cancer, which is of major interest to surgeons.

## 2 METHODS

We conducted a systematic review and meta‐analyses.

### 2.1 Eligibility

Studies in which laparoscopic LAR was compared with open LAR for rectal cancer were eligible. When multiple surgical procedures were included in a study, studies in which over 70% of patients underwent LAR were included. Small studies that included less than 50 patients for each intervention group were excluded. Study design was restricted to RCT, case‐matched study, or cohort study. Both prospective and retrospective studies were included, and the method of randomization or matching was not restricted. The language was restricted to English.

### 2.2 Outcome measures

Short‐term outcomes were the incidence of postoperative overall complications, the incidence of anastomotic leakage, mortality, reoperation rate, length of stay, operative time, estimated blood loss, and rate of positive circumferential resection margins. Long‐term outcomes were 3‐year overall survival (OS), 3‐year disease‐free survival (DFS), and 3‐year local recurrence rate (LRR).

### 2.3 Literature search and study selection

A comprehensive literature search was conducted on June 12, 2019, using PubMed, Scopus, and Cochrane Central Register of Controlled Trials. The search terms used were “rectal cancer”, “anterior resection”, “laparoscopy”, “open”, and related terms (Appendix S1). Duplications were excluded by checking the names of study authors, publication year, and study characteristics such as study design, setting, and period. Two review authors (NH and YF) independently screened the titles and abstracts of studies identified by literature search, and then assessed the full texts of potential eligible articles. Disagreement was resolved by discussion.

### 2.4 Data extraction

The same authors (NH and YF) also independently extracted data from the included studies; the data included study design and setting, number and characteristics of patients, surgical procedure, and short‐ and long‐term outcomes. Each double‐checked the extracted data for the other, and any discordance between them was resolved by discussion. For cohort studies, unadjusted data were extracted to assess the results without adjusting for confounding factors.

### 2.5 Statistical analysis

Data synthesis was carried out using Review Manager 5.3 (Cochrane Collaboration Software, Nordic Cochrane Centre). A random‐effects model was used for all meta‐analyses because of presumed heterogeneity in the surgical quality of LAR across the included studies. The Mantel‐Haenszel method was used for dichotomous variables and inverse‐variance weighting was applied for continuous variables. Risk ratio (RR) with 95% confidence interval (CI) was used for dichotomous variables in a meta‐analysis. Risk difference (RD) was applied instead of RR when a rare outcome was assessed. Mean difference (MD) with 95% CI was used for continuous variables when a single measure was included in a meta‐analysis. The median with range was converted to mean with standard difference (SD) by the method of Hozo et al.^9^ A two‐sided *P*‐value <.05 was considered statistically significant.

## 3 RESULTS

### 3.1 Characteristics of included studies

**Figure 1:**
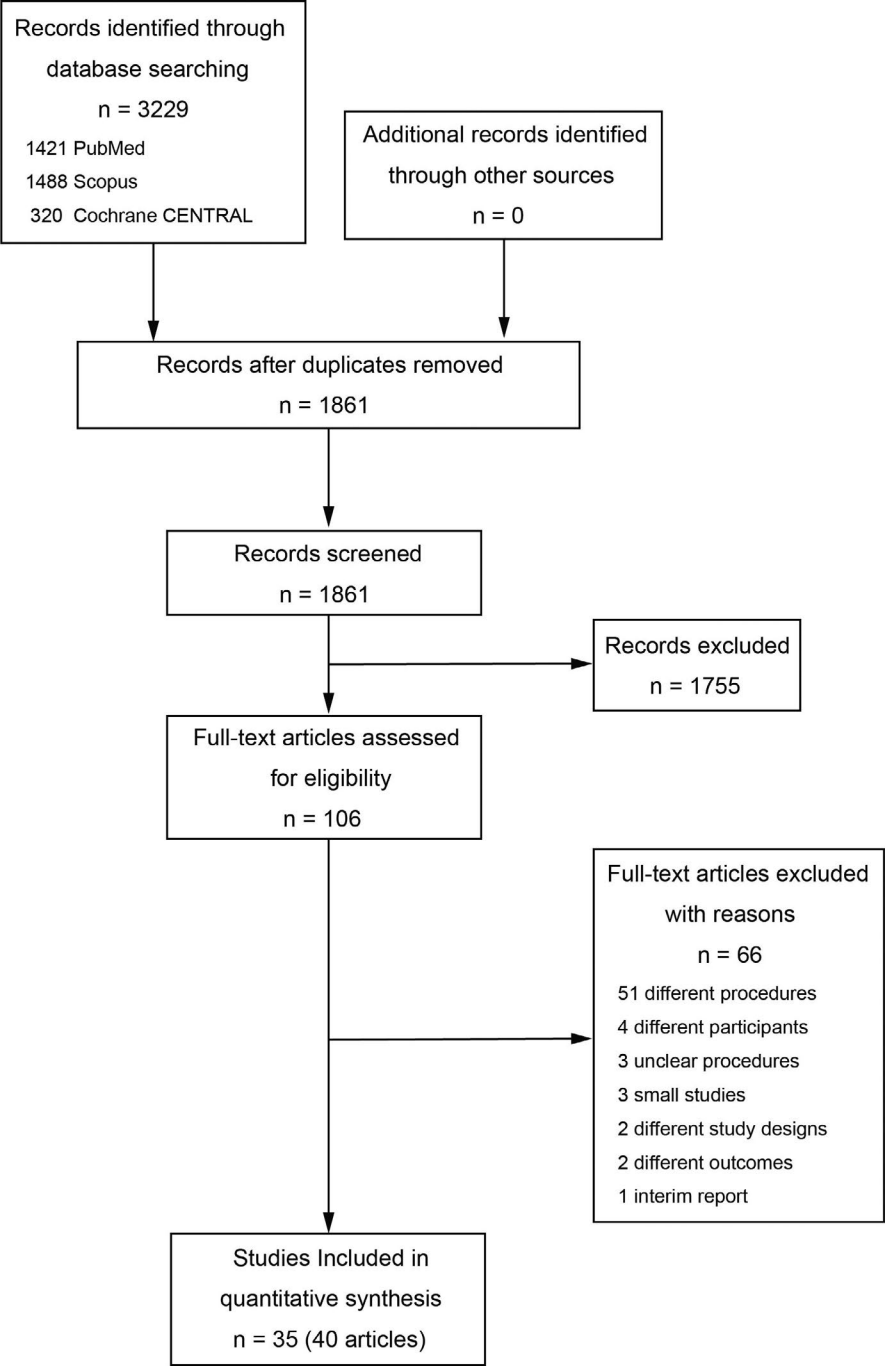
Flow diagram of study selection Central Register of Controlled Trials (CENTRAL)

The comprehensive literature search identified 3229 articles. Of these, 1368 duplications were removed. Screening was conducted for 1861 articles by checking the titles and abstracts for the potential to be included in this review. After screening, the full text of 106 articles was checked to assess whether they met the inclusion criteria. Finally, 35 studies (40 articles) were included in this review (Figure 1)^10^, ^11^, ^12^, ^13^, ^14^, ^15^, ^16^, ^17^, ^18^, ^19^, ^20^, ^21^, ^22^, ^23^, ^24^, ^25^, ^26^, ^27^, ^28^, ^29^, ^30^, ^31^, ^32^, ^33^, ^34^, ^35^, ^36^, ^37^, ^38^, ^39^, ^40^, ^41^, ^42^, ^43^, ^44^, ^45^, ^46^, ^47^, ^48^, ^49^. Nine articles were reported from 4 RCT and were treated as 4 studies^10^, ^11^, ^12^, ^13^, ^14^, ^15^, ^16^, ^17^, ^18^. Included studies comprised 5 RCT, 10 case‐matched studies, and 20 cohort studies. Four studies were conducted internationally and the remaining 31 were reported from 11 countries. One case‐matched study was prospective and the remaining 9 were retrospective. Among cohort studies, 1 was prospective and 19 studies were retrospective (Table 1).

**Table 1:** Patient characteristics

Study		Setting				Prospective/ Retrospective		Study period				Surgical procedures		Patients, n			
		Country		Institution				Start		End				Laparoscopy		Open	
Randomized controlled trials																
ACOSOG^10^, ^11^	International		Multi		Prospective		2008/10		2013/9		LAR, APR, HP, TC		240		222	
ALaCaRT^12^, ^13^	International		Multi		Prospective		2010/3		2014/11		LAR, APR		238		235	
COLAR II^14^, ^15^, ^16^	International		Multi		Prospective		2004/1		2010/5		AR, APR, HP		699		345	
COREAN^17^, ^18^	Korea		Multi		Prospective		2006/4		2009/8		LAR, APR		170		170	
Braga 2007^19^	Italy		Single		Prospective		NR		NR		LAR, APR		83		85	
Case‐matched studies																
da Luz Moreira 2011^20^	USA		Single		Retrospective		1992		2008		LAR, APR		91		91	
de’Angelis 2017^21^	International		Multi		Retrospective		2005/1		2015/12		LAR, APR		52		52	
Guo 2015^22^	China		Single		Retrospective		2007/4		2013/12		LAR, APR		191		191	
Katsuno 2016^23^	Japan		Multi		Prospective		2010/6		2013/2		LAR		209		209	
Koulas 2009^24^	Greece		Single		Retrospective		1998/10		2006/12		AR, ISR, APR		57		60	
Manchon‐Walsh 2019^25^	Spain		Multi		Retrospective		2011		2012		AR,APR, HP		842		517	
Milone 2017^26^	Italy		Multi		Retrospective		2009/1		2015/12		AR, APR		242		235	
Nussbaum 2015^27^	USA		Multi		Retrospective		2010		2011		LAR		6430		6430	
Park 2013^28^	Korea		Single		Retrospective		2003/1		2008/11		AR, ISR, APR		406		406	
Tayar 2018^29^	Brazil		Single		Retrospective		2008		2012		LAR		50		50	
Cohort studies																
Allaix 2016^30^	Italy		Single		Retrospective		1994/4		2005/8		AR, APR		153		154	
Anthuber 2003^31^	Germany		Single		Retrospective		1996/1		2002/3		AR, APR, HP		101		334	
Du 2017^32^	China		Single		Retrospective		2015/1		2017/1		AR		80		70	
Kellokumpu 2012^33^	Finland		Single		Retrospective		1999/1		2006/12		HAR, LAR, APR		100		91	
Kim 2015^34^	Korea		Single		Retrospective		2002/1		2011/12		AR		131		176	
Laurent 2009^35^	France		Single		Retrospective		1994/1		2006/12		AR, APR, HP		238		233	
Law 2009^36^	China		Single		Retrospective		2000/6		2006/12		AR, APR, HP		111		310	
Lee 2013^37^	Korea		Single		Retrospective		2001/6		2008/12		LAR		80		80	
Li 2010^38^	China		Multi		Prospective		2005/6		2007/6		AR, APR		65		70	
Li 2011^39^	China		Single		Retrospective		2000/1		2005/6		AR, APR		113		123	
Li 2015^40^	China		Single		Retrospective		2003/1		2008/12		AR, APR		129		152	
Liu 2016^41^	China		Single		Retrospective		2011		2013		AR, APR		84		65	
Mohamed 2014^42^	China		Single		Retrospective		2000/1		2011/12		AR, APR, HP		470		593	
Pan 2016^43^	China		Single		Retrospective		2009/1		2012/12		AR, APR		85		102	
Staudacher 2007^44^	Italy		Single		Retrospective		1998/1		2005/9		AR		108		79	
Strouch 2013^45^	USA		Single		Retrospective		2005/1		2011/6		LAR		75		75	
Wu 2018^46^	China		Single		Retrospective		2009/1		2013/12		LAR, APR, HP		277		614	
Yang 2013^47^	China		Single		Retrospective		2010/5		2012/5		LAR, APR		87		90	
Zhang 2019^48^	China		Single		Retrospective		2008/1		2011/12		AR, CAA		112		116	
Zhou 2014^49^	China		Single		Retrospective		2005/1		2008/1		LAR, APR		57		65	

Abbreviations: APR, abdominoperineal resection; AR, anterior resection; CAA, colo‐anal anastomosis; HAR, high anterior resection; HP, Hartmann’s procedure; ISR, intersphincteric resection; LAR, low anterior resection; NR, not reported; TC, total colectomy.

### 3.2 Short‐term outcomes

#### 3.2.1 Incidence of overall postoperative complications

Twenty‐four studies with 9881 patients including 4 RCT with 2012 patients, 6 case‐matched studies with 2939 patients, and 14 cohort studies with 4930 patients reported on the incidence of overall complications and were included in a meta‐analysis stratified by study design.

**Figure 2:**
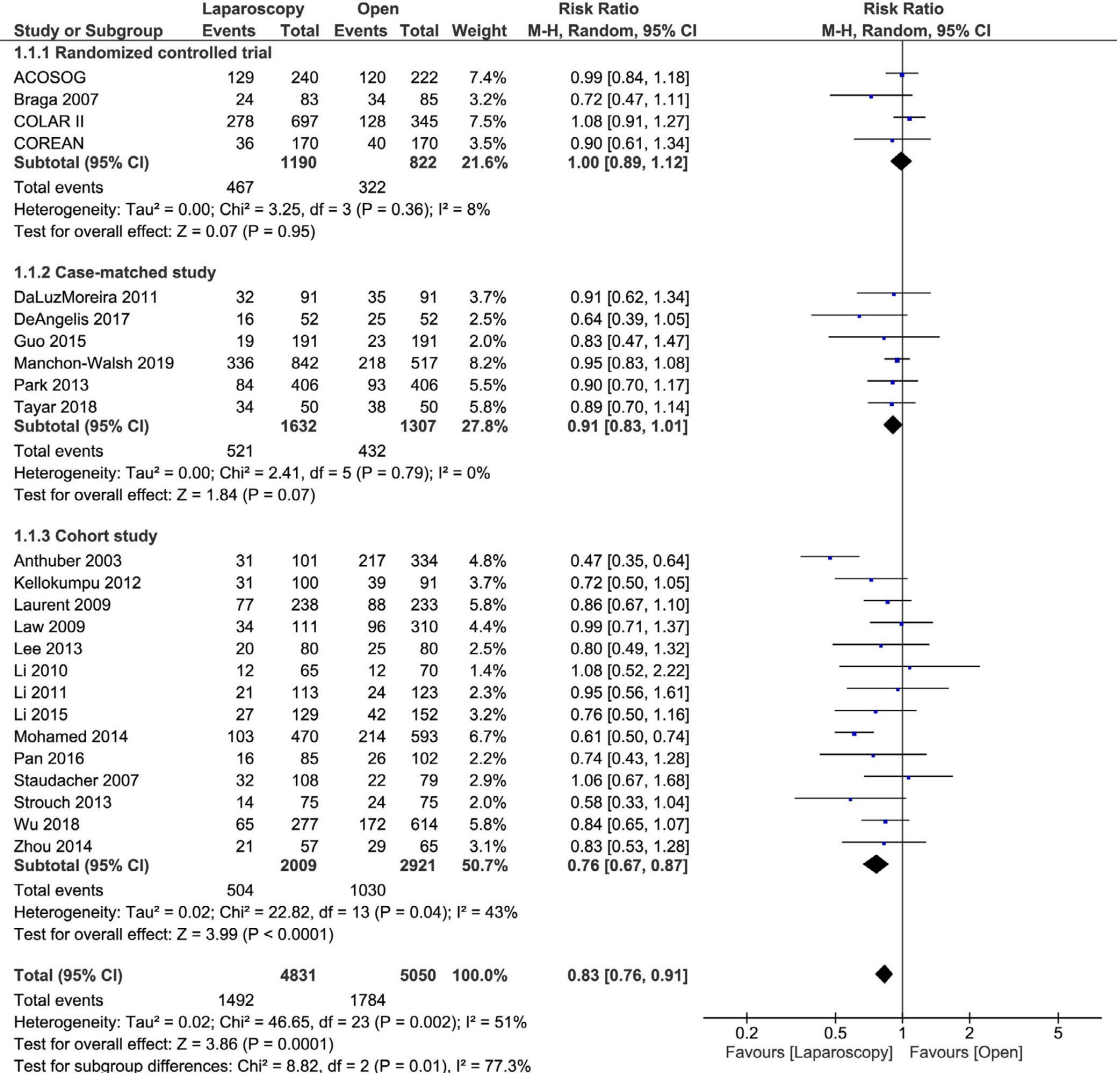
Results of meta‐analysis stratified by study design: Incidence of postoperative overall complications

There were no significant differences between laparoscopic LAR and open LAR in RCT (RR 1.00, 95% CI 0.89‐1.12, *P* = .95) and case‐matched studies (RR 0.91, 95% CI 0.83‐1.01, *P* = .07). However, laparoscopic LAR had a significantly lower incidence of overall postoperative complications than open LAR in cohort studies (RR 0.76, 95% CI 0.67‐0.87, *P* < .001) (Table 2, Figure 2).

**Table 2:** Summary of meta‐analyses based on study designs

Outcomes	Measures	Randomized controlled trials				Case‐matched studies				Cohort studies			
		Study	Patients	Point estimation	95% CI	Study	Patients	Point estimation	95% CI	Study	Patients	Point estimation	95% CI
		n	n			n	n			n	n		
Short‐term													
Postoperative overall complications	RR	4	2012	1.00	0.89, 1.12	6	2939	0.91	0.83, 1.01	14	4930	0.76	0.67, 0.87
Anastomotic leakage	RR	5	2144	1.11	0.78, 1.57	6	1986	1.05	0.79, 1.39	16	5126	0.98	0.77, 1.24
Mortality	RD	5	2487	−0.00	−0.01, 0.00	7	15 799	−0.00	−0.01, 0.00	12	4645	−0.00	−0.01, −0.00
Reoperation	RR	4	2012	1.07	0.71, 1.61	4	1745	1.21	0.67, 2.19	8	3291	0.82	0.60, 1.13
Length of stay	MD	2	630	−1.83	−5.74, 2.07	7	3056	−2.96	−4.50, −1.42	8	2059	−2.15	−3.38, −0.91
Operative time	MD	3	970	48.13	38.11, 58.14	3	1298	−6.32	−60.17, 47.53	10	2480	13.88	−1.16, 28.92
Estimated blood loss	MD	2	630	−116.84	−234.90, 1.22	3	1298	−64.79	−93.98, −35.60	9	1680	−79.71	−108.05, −51.37
Positive CRMs	RR	4	2163	1.25	0.81, 1.92	4	14 644	0.75	0.65, 0.85	6	1679	1.28	0.80, 2.04
Long‐term													
3‐year OS	RR	3	1834	1.02	0.98, 1.06	6	2956	1.05	0.99, 1.10	11	4367	1.06	1.00, 1.12
3‐year DFS	RR	4	2296	1.03	0.97, 1.09	4	1480	0.99	0.94, 1.05	6	1858	0.98	0.95, 1.01
3‐year LRR	RR	4	2002	1.05	0.66, 1.68	2	994	0.70	0.39, 1.25	7	3243	0.64	0.47, 0.88

Abbreviations: CI, confidence interval; CRM, circumferential resection margin; DFS, disease‐free survival; LRR, local recurrence rate; MD, mean difference; OS, overall survival; RD, risk difference; RR: risk ratio.

#### 3.2.2 Incidence of anastomotic leakage

In total, 27 studies with 9256 patients including 5 RCT with 2144 patients, 6 case‐matched studies with 1986 patients, and 16 cohort studies with 5126 patients reported on the incidence of anastomotic leakage and were included in a meta‐analysis stratified by study design.

There were no significant differences between laparoscopic LAR and open LAR in all three types of study design; RCT (RR 1.11, 95% CI 0.78‐1.57, *P* = .57), case‐matched studies (RR 1.05, 95% CI 0.79‐1.39, *P* = .74), and cohort studies (RR 0.98, 95% CI 0.77‐1.24, *P* = .88) (Table 2, Figure S1).

#### 3.2.3 Mortality

Twenty‐four studies with 22 931 patients including 5 RCT with 2487 patients, 7 case‐matched studies with 15 799 patients, and 12 cohort studies with 4645 patients reported on mortality and were included in a meta‐analysis stratified by study design.

There were no significant differences between laparoscopic LAR and open LAR in RCT (RD −0.00, 95% CI −0.01 to 0.00, *P* = .53). In contrast, laparoscopic LAR showed significantly lower mortality than open LAR in case‐matched studies (RD −0.00, 95% CI −0.01 to −0.00, *P* = .03), and cohort studies (RD −0.01, 95% CI −0.01 to −0.00, *P* = .04) (Table 2, Figure S2).

#### 3.2.4 Reoperation rate

A total of 16 studies with 7048 patients including 4 RCT with 2012 patients, 4 case‐matched studies with 1745 patients, and 8 cohort studies with 3291 patients reported on reoperation rate and were included in a meta‐analysis stratified by study design.

No significant differences were noted between laparoscopic LAR and open LAR in all three types of study design: RCT (RR 1.07, 95% CI 0.71‐1.61, *P* = .76), case‐matched studies (RR 1.21, 95% CI 0.67‐2.19, *P* = .53), and cohort studies (RR 0.82, 95% CI 0.60‐1.13, *P* = .22) (Table 2, Figure S3).

#### 3.2.5 Length of stay

Seventeen studies with 5745 patients including 2 RCT with 630 patients, 7 case‐matched studies with 3056 patients, and 8 cohort studies with 2059 patients reported on length of stay and were included in a meta‐analysis stratified by study design.

There were no significant differences between laparoscopic LAR and open LAR in RCT (MD −1.83, 95% CI −5.74 to 2.07, *P* = .36). Conversely, laparoscopic LAR had significantly shorter length of stay than open LAR in case‐matched studies (MD −2.96, 95% CI −4.50 to −1.42, *P* < .001), and cohort studies (MD −2.15, 95% CI −3.38 to −0.91, *P* < .001) (Table 2, Figure S4).

#### 3.2.6 Operative time

In total, 16 studies with 4748 patients including 3 RCT with 970 patients, 3 case‐matched studies with 1298 patients, and 10 cohort studies with 2480 patients reported on operative time and were included in a meta‐analysis stratified by study design.

Laparoscopic LAR had longer operative time than open LAR in RCT (MD 48.13, 95% CI 38.11 to 58.14, *P* < .001). In contrast, there were no significant differences between laparoscopic LAR and open LAR in case‐matched studies (MD −6.32, 95% CI −60.17 to 47.53, *P* = .82) and cohort studies (MD 13.88, 95% CI −1.16 to 28.92, *P* = .07) (Table 2, Figure S5).

#### 3.2.7 Estimated blood loss

Fourteen studies with 3608 patients including 2 RCT with 630 patients, 3 case‐matched studies with 1298 patients, and 9 cohort studies with 1680 patients reported on estimated blood loss and were included in a meta‐analysis stratified by study design.

There were no significant differences between laparoscopic LAR and open LAR in RCT (MD −116.84, 95% CI −234.90 to 1.22, *P* = .05). However, laparoscopic LAR had significantly less estimated blood loss than open LAR in case‐matched studies (MD −64.79, 95% CI −93.98 to −35.60, *P* < .001) and cohort studies (MD −79.71, 95% CI −108.05 to −51.37, *P* < .001) (Table 2, Figure S6).

#### 3.2.8 Rate of positive circumferential resection margins

Fourteen studies with 18 486 patients including 4 RCTs with 2163 patients, 4 case‐matched studies with 14 644 patients, and 6 cohort studies with 1679 patients reported on the rate of positive circumferential resection margins and were included in a meta‐analysis stratified by study design.

There were no significant differences between laparoscopic LAR and open LAR in RCT (RR 1.25, 95% CI 0.81‐1.92, *P* = .31) and cohort studies (RR 1.28, 95% CI 0.80‐2.04, *P* = .30). However, laparoscopic LAR had a significantly lower rate of positive circumferential resection margins than open LAR in case‐matched studies (RR 0.75, 95% CI 0.65‐0.85, *P* < .001) (Table 2, Figure S7).

### 3.3 Long‐term outcomes

#### 3.3.1 Three‐year OS

A total of 20 studies with 9157 patients including 3 RCT with 1834 patients, 6 case‐matched studies with 2956 patients, and 11 cohort studies with 4367 patients reported on the 3‐year OS and were included in a meta‐analysis stratified by study design.

There were no significant differences between laparoscopic LAR and open LAR in RCT (RR 1.02, 95% CI 0.98‐1.06, *P* = .28) and case‐matched studies (RR 1.05, 95% CI 0.99‐1.10, *P* = .09). In contrast, laparoscopic LAR had a significantly higher 3‐year OS rate than open LAR in cohort studies (RR 1.06, 95% CI 1.00‐1.12, *P* = .04) (Table 2, Figure S8).

#### 3.3.2 Three‐year DFS

Fourteen studies with 5634 patients including 4 RCT with 2296 patients, 4 case‐matched studies with 1480 patients, and 6 cohort studies with 1858 patients reported on the 3‐year DFS rate and were included in a meta‐analysis stratified by study design.

There were no significant differences between laparoscopic LAR and open LAR in all three types of study design: RCT (RR 1.03, 95% CI 0.97‐1.09, *P* = .28), case‐matched studies (RR 0.99, 95% CI 0.94‐1.05, *P* = .76), and cohort studies (RR 0.98, 95% CI 0.95‐1.01, *P* = .24) (Table 2, Figure S9).

#### 3.3.3 Three‐year LRR

In total, 13 studies with 6239 patients including 4 RCT with 2002 patients, 2 case‐matched studies with 994 patients, and 7 cohort studies with 3243 patients reported on the 3‐year LRR and were included in a meta‐analysis stratified by study design.

**Figure 3:**
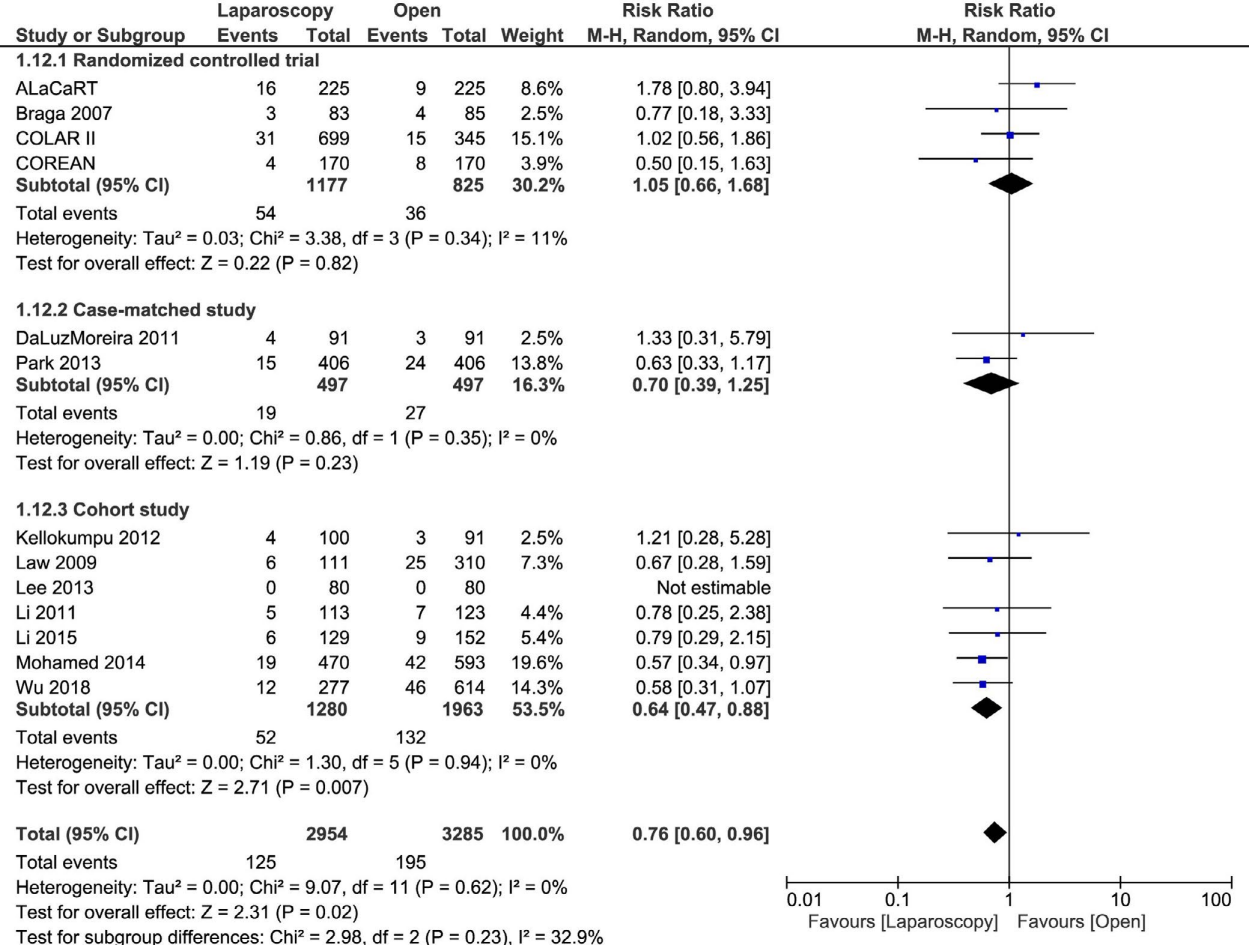
Results of meta‐analysis stratified by study design: 3‐year local recurrence rate

There were no significant differences between laparoscopic LAR and open LAR in RCT (RR 1.05, 95% CI 0.66‐1.68, *P* = .82) and case‐matched studies (RR 0.70, 95% CI 0.39‐1.25, *P* = .23). By contrast, laparoscopic LAR had a significantly lower 3‐year LRR than open LAR in cohort studies (RR 0.64, 95% CI 0.47‐0.88, *P* = .007) (Table 2, Figure 3).

## 4 DISCUSSION

This systematic review and meta‐analysis including 35 studies that compared laparoscopic LAR with open LAR for rectal cancer showed some similarities and differences in findings from the different study designs, particularly RCT and case‐matched studies. Results from cohort studies were used as a reference for unadjusted results potentially affected by confounding factors. We conducted a meta‐analysis for 11 outcomes and found similarities for all outcomes other than operative time and the rate of positive circumferential resection margins between RCT and case‐matched studies. Cohort studies without covariate adjustment tended to overestimate the treatment effect of laparoscopic LAR in the incidence of postoperative overall complications, 3‐year OS, and 3‐year LRR.

Short‐term outcomes were not significantly different between laparoscopic LAR and open LAR in both RCT and case‐matched studies for the incidence of postoperative overall complications, the incidence of anastomotic leakage, and reoperation rate. Meanwhile, significant differences were noted in case‐matched studies but not in RCT regarding mortality, length of stay, and estimated blood loss. However, we deemed these three outcomes similar between RCT and case‐matched studies based on the distribution of 95% CI in forest plots. Thus, the findings of RCT were similar to those of case‐matched studies in terms of 9 short‐term outcomes. However, the number of patients was smaller in RCT than in case‐matched studies and the 95% CI of RCT were wider than those of case‐matched studies in length of stay and estimated blood loss. In addition, the two outcomes can be influenced by the retrospective nature of case‐matched studies. There remains potential difference between RCT and case‐matched studies in these outcomes. Operative time of laparoscopic LAR was significantly longer in RCT but not in case‐matched studies and the rate of positive circumferential resection margins was significantly lower in case‐matched studies but not in RCT. Case‐matched studies might overestimate the treatment effect of laparoscopic LAR in terms of operative time and the rate of positive circumferential resection margins. No significant differences in any long‐term outcomes were noted between laparoscopic LAR and open LAR in both RCT and case‐matched studies. Thus, RCT were similar to case‐matched studies in terms of the majority of outcomes.

We deemed there to be no difference between RCT and case‐matched studies in terms of all outcomes other than operative time and the rate of positive circumferential resection margins. However, we found that the estimated treatment effect of laparoscopic LAR tended to be larger in case‐matched studies compared with RCT but smaller in case‐matched studies compared with cohort studies in the incidence of overall complications and 3‐year LRR according to the distribution of 95% CI in forest plots. RCT can adjust for all confounding factors, and case‐matched studies can adjust for measurable confounding factors only. In this review, cohort studies were not adjusted for any confounding factors because we extracted unadjusted data from cohort studies. Therefore, the difference in the 95% CI between study designs might be due to the difference in adjustments for confounding factors.

Propensity score matching is a representative matching method that was first reported in 1983^3^. Propensity score matching is now widely used, but has several attendant problems such as insufficient reporting of the details of covariant selection, rate of missing covariates, and matching methods^50^, ^51^. Although there are methodological differences between RCT and case‐matched studies such as patient selection and residual confounding, it remains unclear whether findings of RCT differ from those of case‐matched studies^7^, ^8^. Kuss et al reported that outcomes of RCT were similar to those of case‐matched studies in cardiac surgery^52^. According to Dahabreh et al, case‐matched studies potentially overestimate treatment effects in acute coronary syndrome^53^. In addition, there are currently no reports on the similarities and differences between RCT and case‐matched studies in gastrointestinal surgery. In this study, we dealt with LAR for rectal cancer and showed there to be differences between study designs in some outcomes. It remains unclear whether similar results are found in other gastrointestinal surgeries. Further studies are needed to elucidate the similarities and differences in outcomes between study designs in other gastrointestinal surgeries.

This review highlights two outcomes, the incidence of overall postoperative complications and 3‐year LRR, that could be influenced by the adjustment for covariates. The incidence of overall postoperative complications often comprises multiple complications, some of which are sometimes evaluated subjectively, especially in retrospective cohort studies. The subjective assessment of postoperative complications could explain the difference between study designs in terms of outcomes. Nevertheless, local recurrence is usually evaluated objectively by using imaging modalities such as computed tomography and magnetic resonance imaging. Thus, study design can influence both short‐ and long‐term study outcomes, whether the evaluation is subjective or objective. Case‐matched studies and cohort studies are useful in surgical research because it is often difficult to conduct RCT. However, it is necessary to carefully consider the impact of study design on study outcomes.

The strength of the present review is the large number of studies analyzed to comparatively investigate similarities and differences in findings between RCT and case‐matched studies. This review included 35 studies and investigated numerous outcomes including both short‐ and long‐term outcomes for a single comparison. However, this study has some limitations. The number of studies and patients differed between study designs, with a tendency to be lower in RCT and higher in cohort studies. Also, this review included published data only and did not assess study quality. In this review, we showed the similarities and differences in terms of outcomes among RCT, case‐matched studies, and cohort studies on laparoscopic LAR for rectal cancer. Although further study is required in order for the results of this review to be generalizable, we hope these results help clinicians to better interpret the results of surgical research.

In conclusion, findings from case‐matched studies were similar to those of RCT in laparoscopic LAR for rectal cancer. However, findings of case‐matched studies were sometimes intermediate between those of RCT and unadjusted cohort studies, and case‐matched studies and cohort studies had a potential to overestimate the treatment effect compared with RCT.

## DISCLOSURE

Funding: This review was supported by a grant from Kondou Kinen Medical Foundation.

Conflicts of interest: The authors declare no conflicts of interest for this study.

Author Contribution: All authors contributed to the study concept and design. Literature search and data collection were performed by NH and YF. Statistical analysis was conducted by NH and checked by the other authors. The first draft of manuscript was written by NH and all authors commented on previous version of the manuscript. All authors read and approved the final.

## Supporting information

Fig S1

Fig S2

Fig S3

Fig S4

Fig S5

Fig S6

Fig S7

Fig S8

Fig S9

Supplementary Material

Supplementary Material
