# Supplementary figures and images for: Similarities and differences between study designs in short‐ and long‐term outcomes of laparoscopic versus open low anterior resection for rectal cancer: A systematic review and meta‐analysis of randomized, case‐matched, and cohort studies
Source: Ann Gastroenterol Surg. 2020 Nov 21;5(2):183–93. doi: 10.1002/ags3.12409 (PMC8034685; doi:10.1002/ags3.12409)

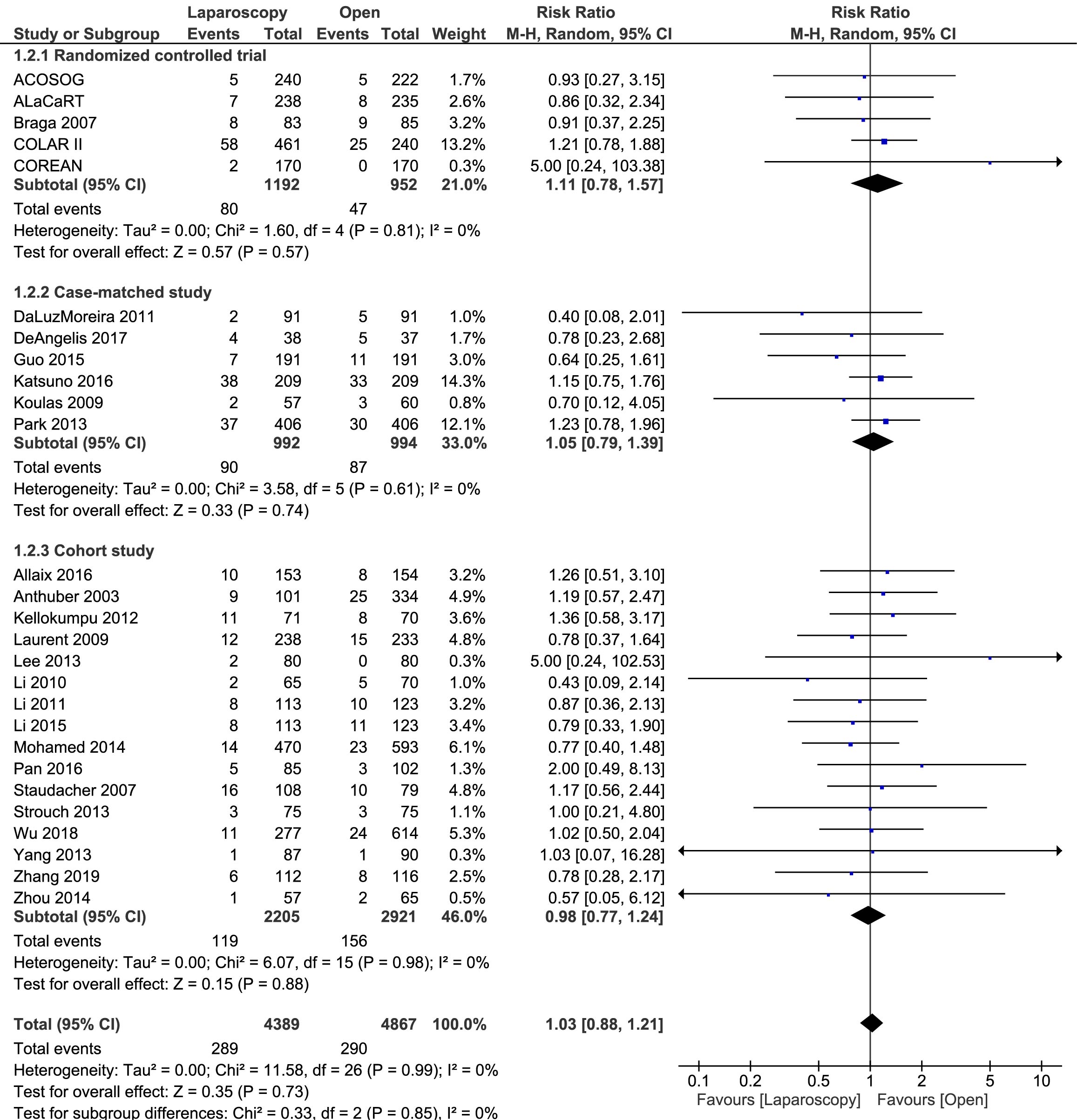

Supplement: Supplementary file 1 — Fig S1 [file AGS3-5-183-s006.jpg]

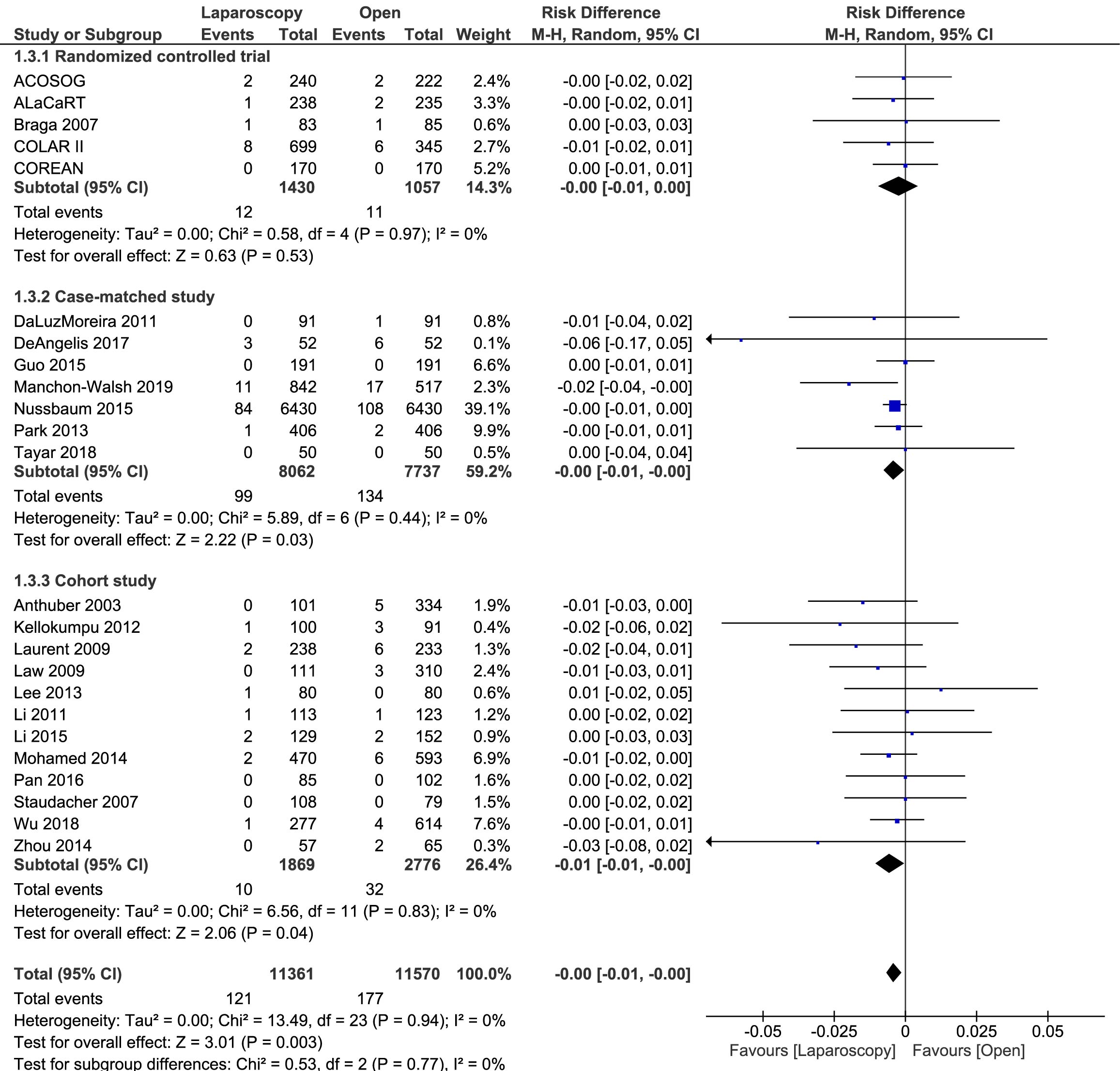

Supplement: Supplementary file 2 — Fig S2 [file AGS3-5-183-s004.jpg]

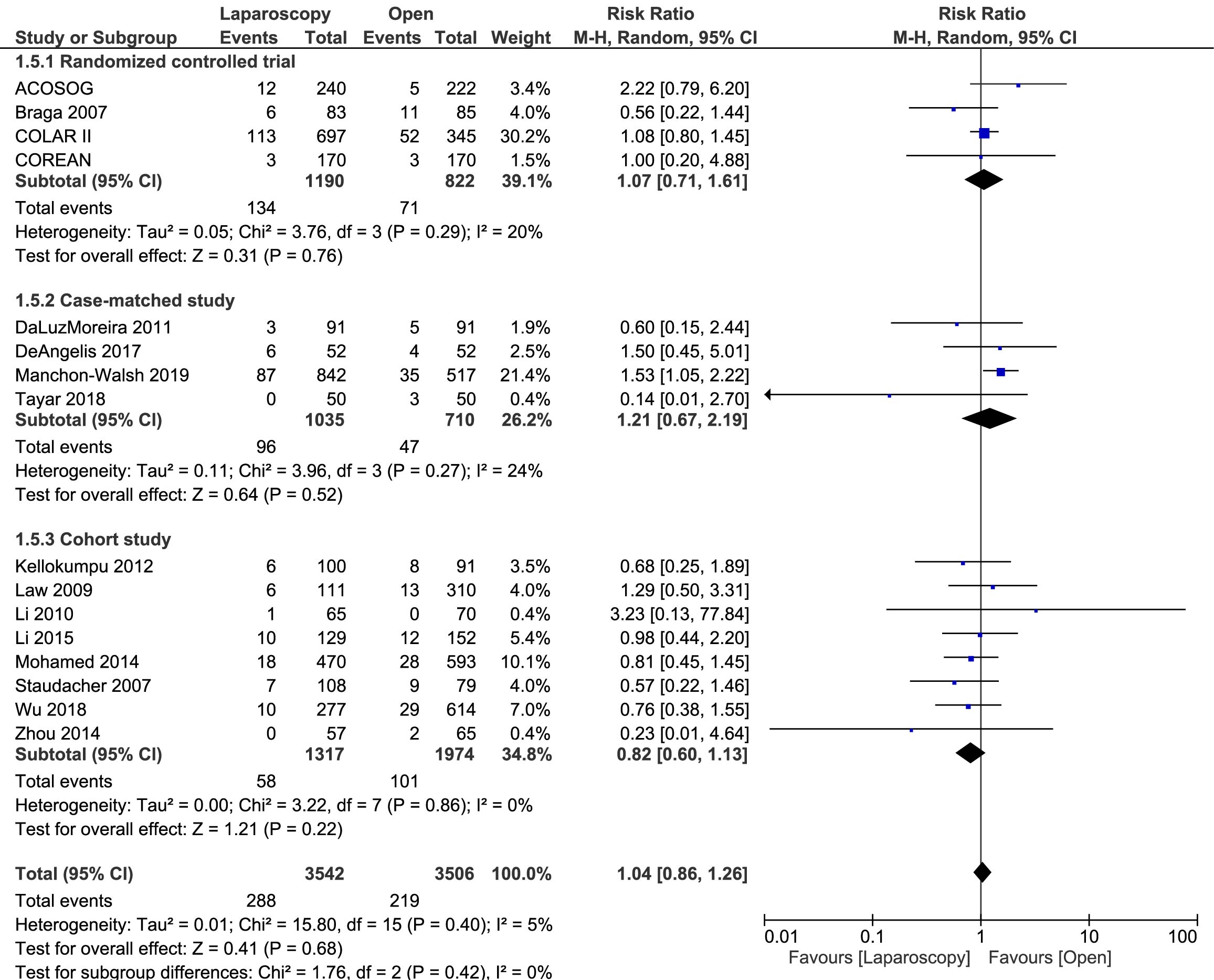

Supplement: Supplementary file 3 — Fig S3 [file AGS3-5-183-s005.jpg]

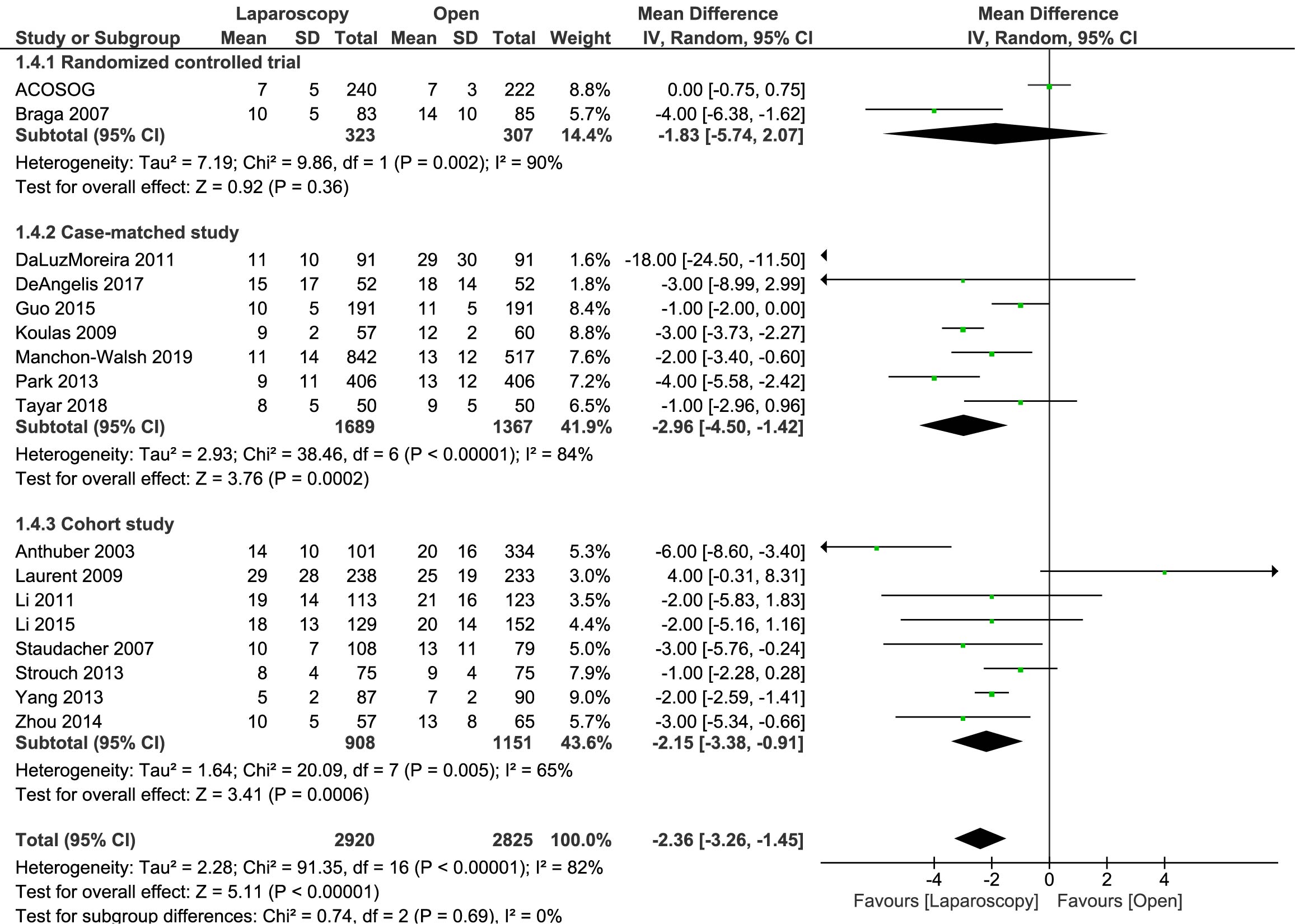

Supplement: Supplementary file 4 — Fig S4 [file AGS3-5-183-s008.jpg]

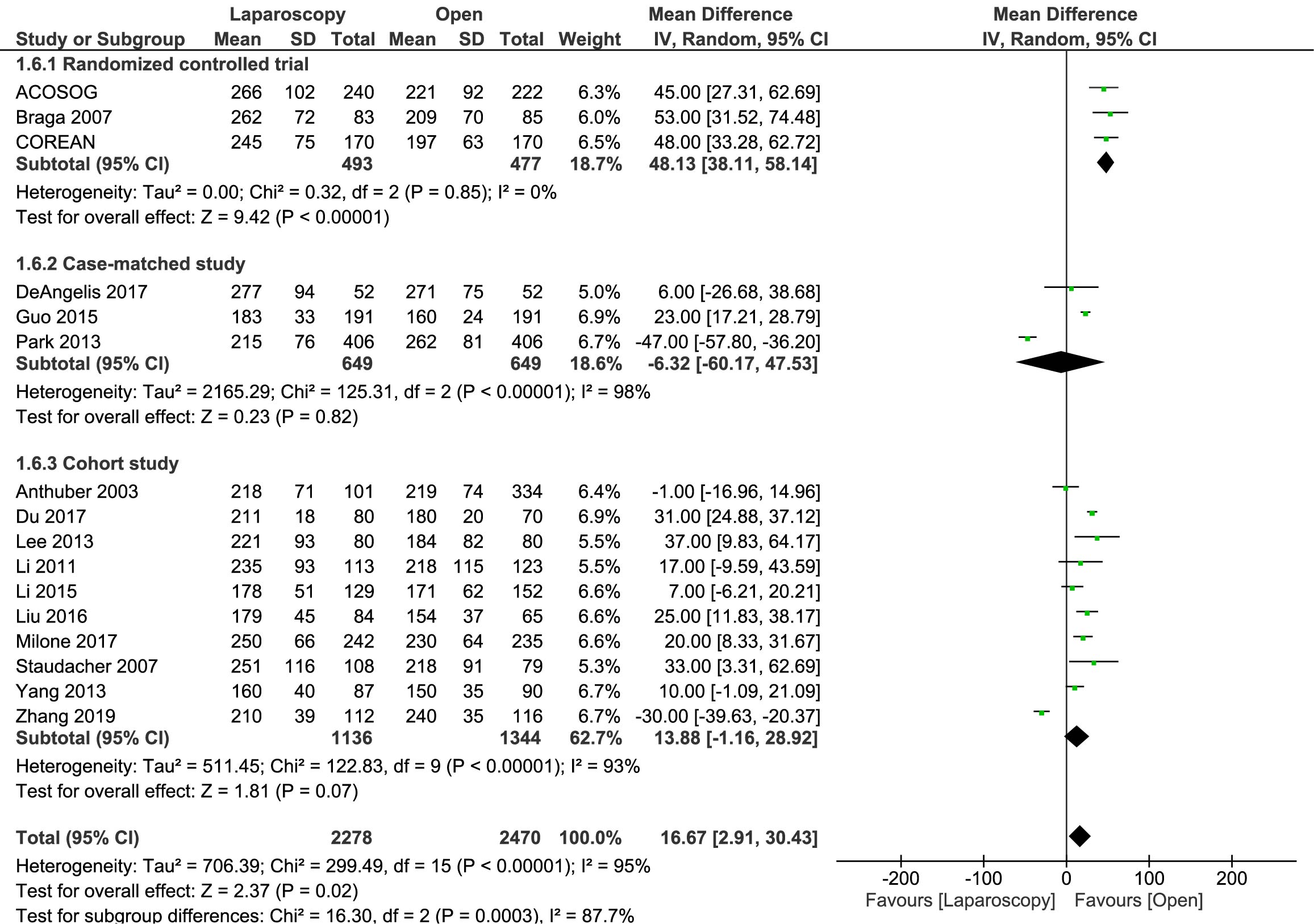

Supplement: Supplementary file 5 — Fig S5 [file AGS3-5-183-s002.jpg]

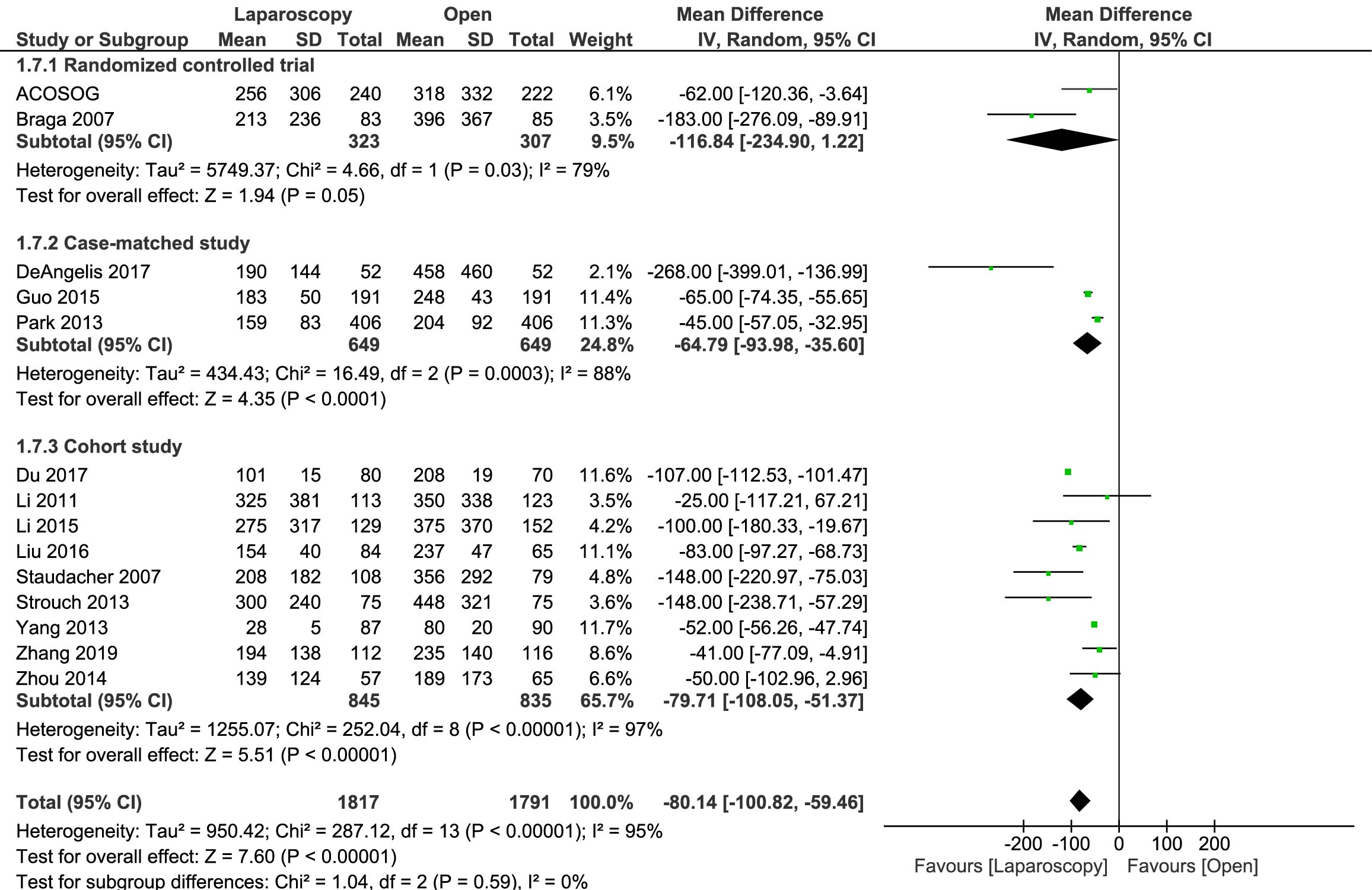

Supplement: Supplementary file 6 — Fig S6 [file AGS3-5-183-s001.jpg]

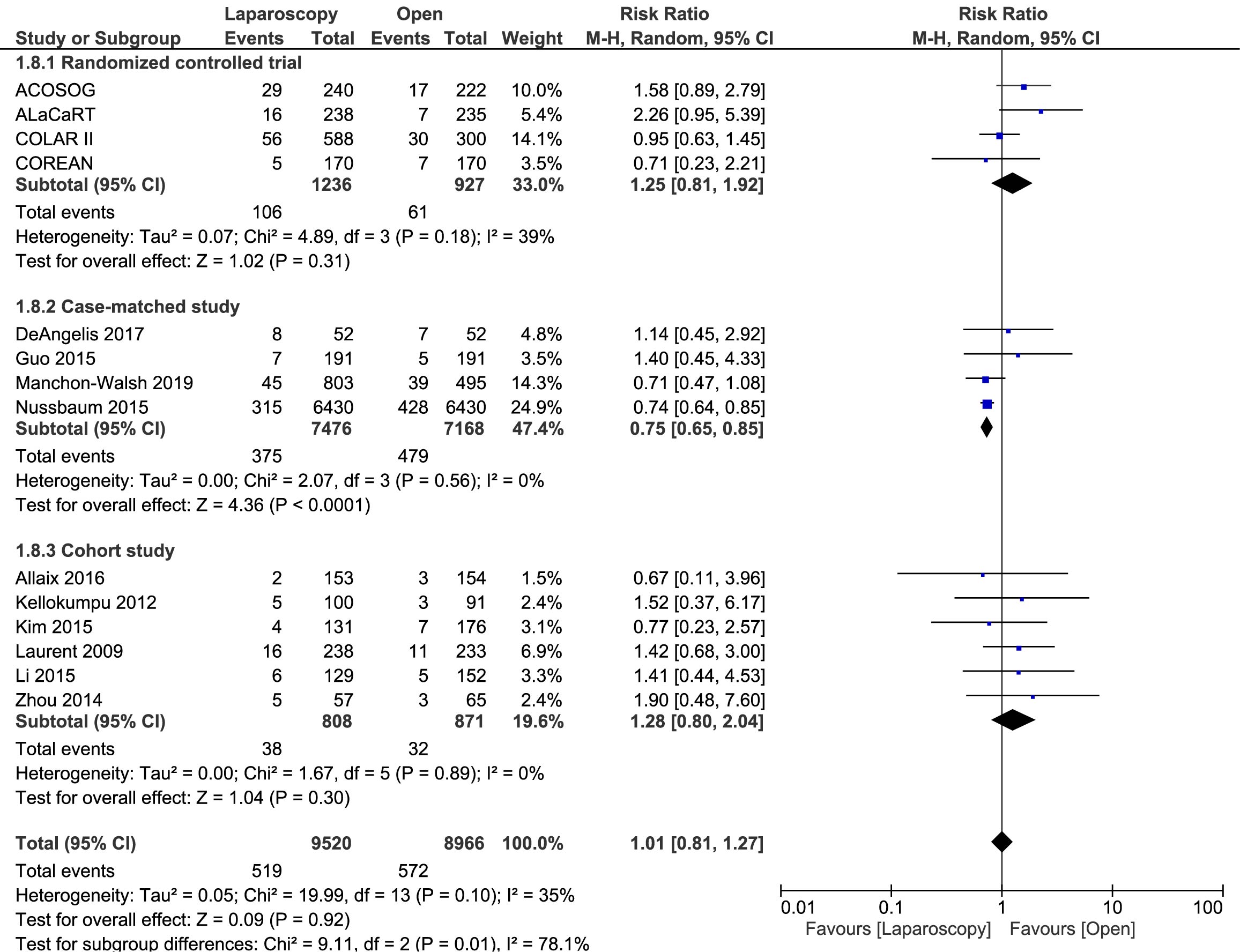

Supplement: Supplementary file 7 — Fig S7 [file AGS3-5-183-s010.jpg]

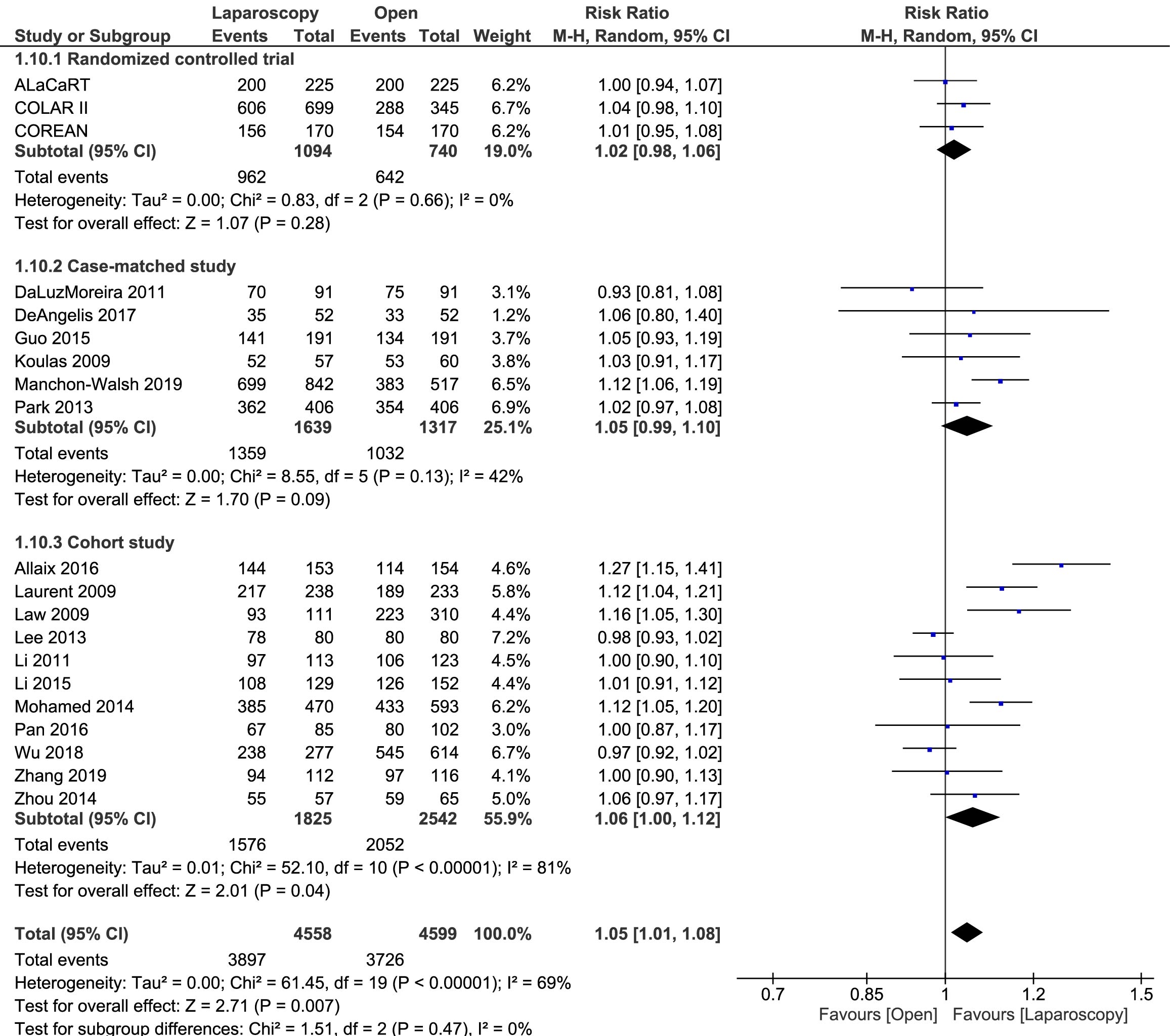

Supplement: Supplementary file 8 — Fig S8 [file AGS3-5-183-s011.jpg]

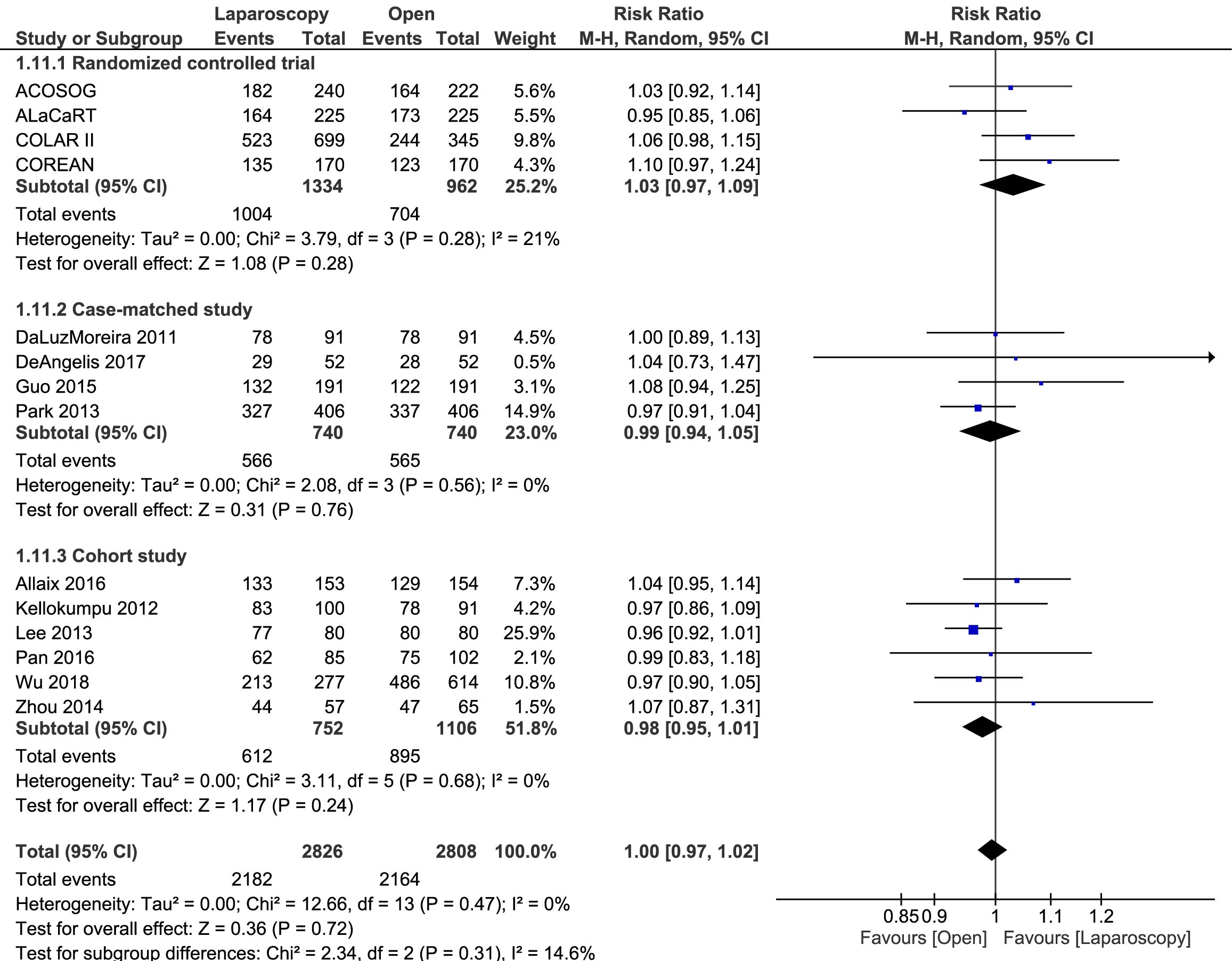

Supplement: Supplementary file 9 — Fig S9 [file AGS3-5-183-s003.jpg]
