# Supplementary material for: Similarities and differences between study designs in short‐ and long‐term outcomes of laparoscopic versus open low anterior resection for rectal cancer: A systematic review and meta‐analysis of randomized, case‐matched, and cohort studies
Source: Ann Gastroenterol Surg. 2020 Nov 21;5(2):183–93. doi: 10.1002/ags3.12409 (PMC8034685; doi:10.1002/ags3.12409)
Supplement: Supplementary file 10 — Supplementary Material [file AGS3-5-183-s007.docx]

**Search strategy for PubMed**

#1 "Rectal neoplasms"[MeSH Terms]

#2 rectum[Tiab] OR rectal[Tiab]

#3 neoplasm[Tiab] OR cancer[Tiab] OR tumor[Tiab] OR carcinoma[Tiab]

#4 #2 AND #3

#5 #1 OR #4

#6 “Laparoscopy”[MeSH Terms]

#7 laparoscopy[Tiab] OR laparoscopic[Tiab]

#8 #6 OR #7

#9 “Laparotomy”[MeSH Terms]

#10 laparotomy[Tiab] OR open[Tiab]

#11 #9 OR #10

#12 “Proctocolectomy, Restorative” [MeSH Terms]

#13 surgery[Tiab] OR operation[Tiab] OR resection[Tiab] OR proctectomy[Tiab] OR “anterior resection”[Tiab] OR “mesorectal excision”[Tiab] OR “mesorectum excision”[Tiab]

#14 #12 OR #13

#15 #5 AND #8 AND #11 AND #14
