# Supplementary material for: Similarities and differences between study designs in short‐ and long‐term outcomes of laparoscopic versus open low anterior resection for rectal cancer: A systematic review and meta‐analysis of randomized, case‐matched, and cohort studies
Source: Ann Gastroenterol Surg. 2020 Nov 21;5(2):183–93. doi: 10.1002/ags3.12409 (PMC8034685; doi:10.1002/ags3.12409)
Supplement: Supplementary file 11 — Supplementary Material [file AGS3-5-183-s009.docx]

**Fig. S1** Results of meta-analysis stratified by study design: incidence of anastomotic leakage

**Fig.S2** Results of meta-analysis stratified by study design: mortality

**Fig. S3** Results of meta-analysis stratified by study design: reoperation rate

**Fig. S4** Results of meta-analysis stratified by study design: length of stay

**Fig. S5** Results of meta-analysis stratified by study design: operative time

**Fig. S6** Results of meta-analysis stratified by study design: estimated blood loss

**Fig. S7** Results of meta-analysis stratified by study design: rate of positive circumferential resection margins

**Fig. S8** Results of meta-analysis stratified by study design: 3-year overall survival

**Fig. S9** Results of meta-analysis stratified by study design: 3-year disease-free survival

**Appendix1** Search strategy for PubMed
